# Biosecurity Concept: Origins, Evolution and Perspectives

**DOI:** 10.3390/ani12010063

**Published:** 2021-12-28

**Authors:** Véronique Renault, Marie-France Humblet, Claude Saegerman

**Affiliations:** 1 Research Unit in Epidemiology and Risk Analysis Applied to Veterinary Sciences (UREAR-ULiege), Fundamental and Applied Research for Animal Health (FARAH) Centre, Faculty of Veterinary Medicine, University of Liege, 4000 Liege, Belgium; vrenault@uliege.be; 2 Biosafety and Biosecurity Unit, Department of Occupational Safety and Health, University of Liege, 4000 Liege, Belgium; mfhumblet@uliege.be

**Keywords:** biosafety, biosecurity, one health

## Abstract

**Simple Summary:**

The term biosecurity first referred to biological weapons and bio-terrorism. It is now used in diverse sectors including biological laboratory risks and infectious disease prevention in both animal and public health. Therefore, several definitions and understandings of the term biosecurity co-exist. This commentary aims to describe the evolution of the biosecurity concept over the years and discuss its possible future.

**Abstract:**

Originally used in reference to the management of biological weapons and bio-terrorism, the term biosecurity was first used in the agricultural sector in the 1980s as “the sum of risk management practices in the defence against biological threats”. This term was then taken up in different strategic documents of different organisations, so multiple definitions and understandings co-exist. This short communication reviews the origins and evolution of the biosecurity concept and discusses the future perspectives of biosecurity in regard to the One Health Approach and the changing environment.

## 1. Introduction

Over the years, infectious diseases have had a huge impact on both animal and public health. The 19th century saw the foundation of epidemiology as a scientific discipline with the studies of John Snow during the 1845 cholera outbreak in London [1]. Those studies identified contaminated water as the source of the disease, the main contamination pathways and deduced the hygienic measures to be taken in order to control it. The 19th century also witnessed the development of the first vaccines. The discovery of antibiotics in the 20th century was a major step towards the control and eradication of some major infectious diseases. Since then, the era of modern medicine has led to the development of major public health improvements and epidemiological knowledge. The incidence of infectious diseases was largely reduced and several diseases were regionally eradicated. In the 1960s, medical and public health professionals were convinced that infectious diseases “were gradually going to disappear under the combined influence of hygiene, antibiotics, and vaccines” [2]. Indeed, the development of the biomedical sciences in the 20th and 21st centuries led to major successes in disease control and prevention such as the eradication of small pox and rinderpest, as well as the regional eradication and control of other infectious diseases. Nevertheless, the last decades saw the (re) emergence of infectious diseases, 61% of which were zoonotic, and 75% of new human pathogens originating from animals [3,4]. The drivers of the(re)emergence and spread of infectious diseases include globalization and demographic changes, and new agricultural production and food processing technologies have increased international movements of goods, animals and persons.

Therefore, a shift from curative to preventive medicine has been observed over the years. If the first practitioners in both medical and animal health were mainly asked to treat diseases, the necessity and benefit of preventing them would not be a modern concept [5,6]. Preventive medicine implies a proper knowledge of the aetiology and epidemiology of infectious diseases. Nevertheless, several examples of preventive measures have been found that date to 2000 bc [5], long before microorganisms such as moulds and bacteria were discovered in 1676 [7]. Over time, different protective measures against infectious diseases were identified and proven effective based on observation and trial and error. We could mention, as key examples, the measures developed in the 14th century in Italy and southern France against the plague (e.g., sanitary cordon, observation and isolation facilities, and disinfection procedures) [6], and the smallpox variolation practice that was reported as early as 1670 [8]. Due to the progress in sciences and epidemiology, many measures to prevent and control these diseases were identified and promoted; the biosecurity concept emerging as preventive medicine became a key element of both public and animal health.

## 2. Biosecurity: The Initial Concept and Existing Definitions

**Figure 1:**
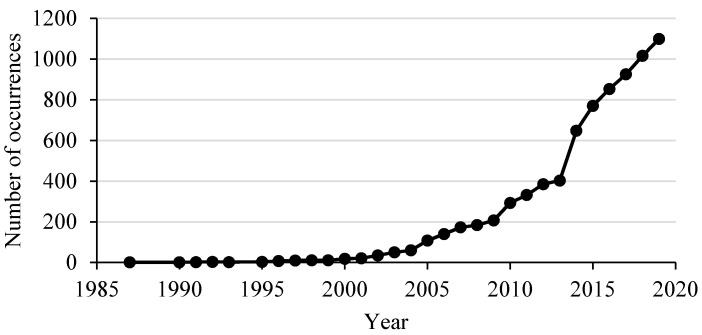
Number of occurrences of “biosecurity” in PubMed.gov.

Originally, “biosecurity” was mainly used in defence regarding the control of biological weapons. This first definition of biosecurity still appears on some official documents and websites. As an example, the Belgian biosafety server defines biosecurity as “the prevention of misuse through loss, theft, diversion or intentional release of pathogens, toxins and any other biological materials” (https://www.biosafety.be/content/biosecurity, accessed on 15 December 2021). Nevertheless, in the 1980s ‘’biosecurity” started to be used regarding animal health and production systems; it was defined by the U.S. Association of State Departments of Agriculture as “the vital work of strategy, efforts and planning to protect human, animal and environmental health against biological threats”. The first citation of “biosecurity” in PubMed was recorded in 1987. Its general uptake increased slowly with an average of five publications including the term “biosecurity” per year in the 1990s, 127 from 2000 to 2010 and 680 from 2011 to 2020 (Figure 1) [9].

The primary goal of biosecurity is to protect against risks posed by diseases and organisms. The primary tools of biosecurity are exclusion, eradication and control, supported by expert system management, practical protocols, and the rapid and efficient securing and sharing of vital information. Although frequently used in the scientific literature as being similar to biosafety [10], biosecurity is different. Biosafety is complementary to biosecurity and refers to “the implementation of laboratory practices and procedures, specific construction features of laboratory facilities, safety equipment, and appropriate occupational health programs when working with potentially infectious microorganisms and other biological hazards” [11]. For example, biosafety is defined in Belgium as “the safety for human health and the environment, including the protection of biodiversity, during the use of genetically modified organisms (GMOs) or micro-organisms (GMMs), and during the contained use of pathogenic organisms for humans” (https://www.biosafety.be/content/belgian-regulatory-framework-biosafety, accessed on 15 December 2021).

Over the years, the concept of biosecurity has been largely adopted by a number of countries and incorporated in several strategic documents of different sectors. In the animal health and production sector, biosecurity is defined as “a set of management and physical measures designed to reduce the risk of introduction, establishment and spread of animal diseases, infections or infestations to, from and within an animal population” (https://www.oie.int/fileadmin/Home/eng/Health_standards/tahc/2018/en_glossaire.htm, accessed on 15 December 2021). This definition is referred to in the Animal Health Law (Regulation (EU) 2016/429). Different definitions of biosecurity, therefore, co-exist as attempts are made to propose a unified definition as “the strategies to assess and manage the risk of infectious diseases, quarantine pests, invasive alien species, living modified organisms, and biological weapons” [12].

## 3. The Current Understanding and Definition of Biosecurity

For the UN’s Food and Agriculture Organization (FAO) and World Health Organization (WHO), “biosecurity is a strategic and integrated concept that encompasses the policy and regulatory frameworks (including instruments and activities) that analyse and manage risk in food safety, public health, animal life and health, and plant life and health, including associated environmental risk” [13]. Since 2007, biosecurity has been included as a key element in the European Union Animal Health Strategy [14] and in the country preparedness plan of the European Centre for Disease Prevention and Control (ECDC) [15]. Earlier, it was enclosed in the International Health Regulation adopted by the WHO in 2005 [16].

**Figure 2:**
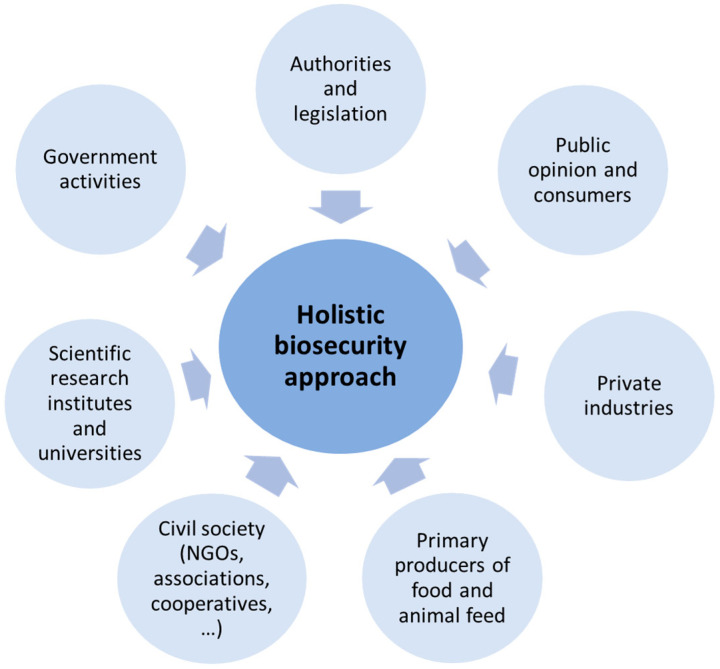
Sector interests that are important to a holistic approach of biosecurity (Adapted from the FAO, 2007 [18]).

Biosecurity includes all measures to prevent the introduction of pathogens (bio-exclusion) and reduce the spread of pathogens (bio-containment) [17]. As part of the One Health concept, biosecurity is particularly important as it includes the prevention of the spread to humans, animals, plants and the environment. It is therefore a holistic and integrated approach, which considers the interactions among different stakeholders and sectors, as presented in Figure 2. A stringent biosecurity level, therefore, minimises the impact of infectious diseases on public, animal and plant health, as well as the economy, the environment and society in general. The FAO and WHO definition of biosecurity, which includes these aspects, is therefore appropriate and should be considered by the other actors as a reference to emphasise the importance of biosecurity, not only for animal health but also for public and environmental health.

The different kinds of hazards that biosecurity targets relate to different sectors (e.g., food safety and human, animal and plant health) with special attention paid to zoonoses, biological weapons and invasive alien species. Therefore, based on the sector, the risk-and-hazard definitions to be addressed are multiple (see examples in Table 1).

## 4. Perspectives and Conclusions

**Figure 3:**
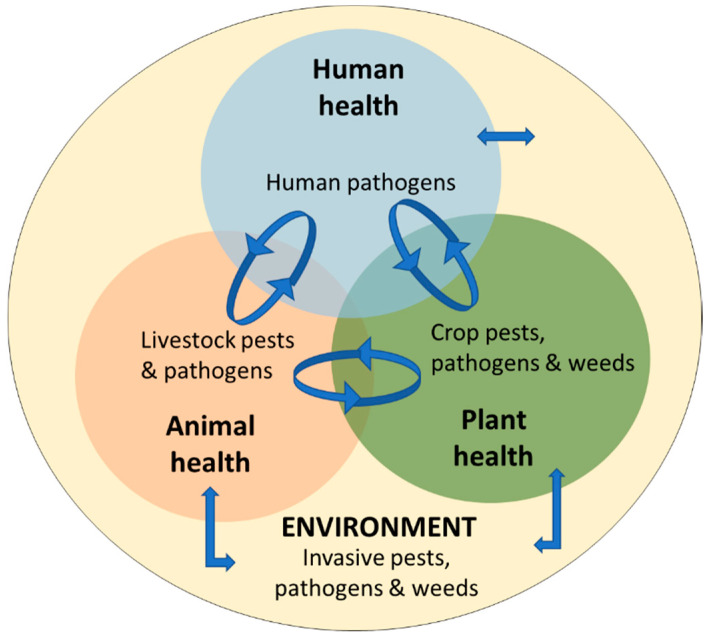
Schematic representation of the links among human, animal, plant, and environmental health (adapted from Hulme, 2020 [19]).

The OIE definition mentions the risk of “infections or infestations to, from and within an animal population”. This definition does not explicitly mention links with public and environmental health, which can then be forgotten or undermined. The FAO and WHO definition already clarifies these aspects, ensuring links with farm biosecurity and the proper consideration of public and environmental health. Due to the international context, the risks of infectious diseases to animals, humans, plants and the environment, as illustrated in Figure 3, are increasing. Therefore, the importance of biosecurity as a key element of the One Health concept will have to be developed further and addressed properly. 

In the future, the importance of biosecurity in mitigating the risks for animal and public health and environmental contamination will have to be further developed and taken into account. The concept of “One biosecurity” was already introduced in a recent publication [19] as “a unified concept to integrate human, animal, plant, and environmental health”. The implementation of this broader concept of biosecurity will need a strengthened collaboration and interaction among the different sectors at all levels, which represents a major challenge. The success will greatly depend on the political, legislative and collaborative structures that will prevail and their capacity to integrate the necessary elements for an effective intersectoral collaboration In all official communications or programmes mentioning biosecurity, special attention is required to ensure that its definition is comprehensive and not limited to animal or public health.

## Figures and Tables

**Table 1:** Examples of different types of hazards applicable to different biosecurity sectors (Adapted from FAO, 2007 [18]).

Biosecurity Sector	Hazard Definition
Food safety	A biological, chemical or physical agent in, or condition of, food with the potential to cause an adverse health effect
Zoonoses	A biological agent that can be transmitted naturally between wild or domestic animals and humans
Animal health	Any pathogenic agent that could produce adverse consequences on animal health
Plant health (or pest)	Any species, strain or biotype of plant, animal or pathogenic agent injurious to plants or plant products
“Biosafety” in relation to plants and animals	A living modified organism (LMO) that possesses a novel combination of genetic material obtained through the use of modern biotechnology that is likely to have adverse effects on the conservation and sustainable use of biological diversity, taking also into account risks to human health (Cartagena Protocol on Biosafety).
“Biosafety” in relation to food	A recombinant DNA organism directly affecting or remaining in a food that could have an adverse effect on human health
Invasive alien species	An invasive alien species outside its natural past or present distribution whose introduction and/or spread threatens biodiversity

## Data Availability

No new data were created or analysed in this study. Data sharing is not applicable to this article.
